# MiR-205 as predictive biomarker and adjuvant therapeutic tool in combination with trastuzumab

**DOI:** 10.18632/oncotarget.24723

**Published:** 2018-06-15

**Authors:** Alessandra Cataldo, Claudia Piovan, Ilaria Plantamura, Elvira D’Ippolito, Simone Camelliti, Patrizia Casalini, Marta Giussani, Olivier Déas, Stefano Cairo, Jean-Gabriel Judde, Elda Tagliabue, Marilena V. Iorio

**Affiliations:** ^1^ Start Up Unit, Experimental Oncology Department, Fondazione IRCCS Istituto Nazionale dei Tumori, Milan, Italy; ^2^ Molecular Targeting Unit, Experimental Oncology Department, Fondazione IRCCS Istituto Nazionale dei Tumori, Milan, Italy; ^3^ Xentech, Genopole Campus 3, Evry, Paris

**Keywords:** microRNA, breast cancer, HER2-3, trastuzumab, biomarkers

## Abstract

Trastuzumab is the standard treatment for HER2+ breast cancer (BC) patients, and even though it significantly improved their clinical outcome, 50% of them do not benefit from this drug and disease recurs, underlining the need of reliable predictive biomarkers and new therapeutic strategies. Strikingly, despite all the molecular analyses performed to identify the escape mechanisms behind this resistance, it still represents a question point. MiRNAs have been correlated with occurrence and progression of human cancer, and their potential as clinical tools has emerged in the last years. We previously reported that oncosuppressive miR-205 targets HER3, thus increasing the responsiveness to TKIs lapatinib and gefitinib in preclinical models.

Here we demonstrate that HER3 inhibition by miR-205 ectopic expression or siRNA-mediated silencing improves the responsiveness to Trastuzumab *in vitro* in HER2+ BC cell lines, and that this effect is exerted through impairment of AKT-mediated pathway. Moreover, evaluating a series of 52 HER2+ BC patients treated with adjuvant Trastuzumab, we observed that higher miR-205 expression is significantly associated with better outcome (disease-free survival).

In summary, our data indicate that miR-205 could predict Trastuzumab efficacy and that its modulation might be useful as adjuvant treatment to improve the response to the drug.

## INTRODUCTION

Breast cancer (BC) is the first most common malignant disease and the second leading cause of cancer mortality in women. In 2014, the estimated new cases and deaths due to BC in the United States were 232,670 and 40,000, respectively [1]. The subgroup showing amplification and/or overexpression of HER2 oncogene represents approximately 20% of BC, and associates with aggressiveness and poor prognosis [2]. Trastuzumab is the standard-of-care treatment for these patients, and significantly improved their clinical outcome. Even so, 50% of patients do not benefit from this drug and disease recurs [3]. Moreover, even the introduction of dual anti-HER2 combinations have generated unsatisfactory results, since a still high percentage of patients develop primary or acquired resistance. Resistance to standard therapies is indeed a major issue in medical oncology, and has two main consequences: the need of reliable predictive biomarkers to treat only patients who will actually benefit from that treatment, avoiding detrimental side effects and delay in starting new treatments; and the need of new therapeutic strategies.

Retrospective studies have indicated that tumor dependence on HER2 [4] or the presence of immune infiltrate [5] might represent predictive biomarkers of Trastuzumab efficacy. However, elucidating the molecular pathways involved in Trastuzumab resistance has been difficult due to the variety of mechanisms of action of this drug [6]. Multiple mechanisms have been proposed, as the PI3K/AKT/PTEN pathway deregulation, reduced receptor-antibody binding affinity, and increased signaling via alternative receptors [7, 8]. However, final validations based on analyses of human samples have been limited and are not entirely reproducible. Globally, despite all the molecular analyses performed and the efforts to identify the escape mechanisms behind this resistance, it still represents a question point.

MiRNAs have been correlated with occurrence and progression of human cancer, including BC [9], and their potential as clinical tools has recently emerged [10]. In the effort to elucidate the mechanisms of resistance to anti-HER2 therapies, the approach mainly applied to date has been the study of miRNAs differentially expressed in resistant *versus* parental cells [11, 12]. A similar strategy has been the identification of miRNAs modulated following Trastuzumab treatment. However, only few reports have investigated the effects of miRNA modulation *in vivo* in xenograft models in combination with Trastuzumab [13, 14]. Moreover, even fewer reports have evaluated these miRNAs in clinical specimens of HER2+ BC and associated their expression with Trastuzumab response: for instance, higher expression of miR-21 in the primary tumor of patients treated with neoadjuvant Trastuzumab has been correlated with worse response [14, 6]. Notably, the same miRNA did not associate with outcome in patients treated in adjuvant setting [15]; however this observation was made only on 16 patients and mechanisms involved in the effects of a specific compound in adjuvant setting might be different. Surprisingly, serum circulating miR-21 did not associate with PR (pathological response) in patients enrolled in the GeparQuinto trial (neo-adjuvant chemotherapy plus Trastuzumab or lapatinib) [16]; however levels of serum miRNAs might be affected by other regulatory mechanisms. In summary, a validated predictive signature of response to Trastuzumab has not been defined yet; moreover, the investigation of miRNAs potentially involved in the responsiveness to the drug and their possible clinical application as adjuvant therapeutic tools is still in its infancy.

We previously reported that oncosuppressive miR-205 targets HER3 and impairs the downstream AKT-mediated survival pathway, thus increasing the responsiveness to TKIs lapatinib and gefitinib in preclinical models [17]. Since HER3 activation is an escape mechanism also involved in Trastuzumab resistance, miR-205 could be a promising predictive biomarker of responsiveness to this treatment as well as a possible tool for a combined therapy.

## RESULTS

### MiR-205 as adjuvant tool in combination with Trastuzumab

To evaluate the effect of miR-205 on the responsiveness to Trastuzumab, HER2+ BC cell line SKBr3 has been transfected with 100 nΜ miR-205 precursor or negative control (miR-205 and miR-neg) for 24 h, and treated with 1 ug/ml Trastuzumab for the following 48 h. In parallel, as phenocopy experiment, HER3 has been silenced with a siRNA sequence or siRNA control (si-HER3 and si-CTR) with the same schedule of transfection and treatment. Once collected, cells have been first analyzed for miR-205 expression, to evaluate the transfection efficiency (Supplementary Figure 1). Western Blot analyses have been performed to verify the down-regulation of HER3 following miR-205 or siRNA transfection, and to assess the receptor activation and the modulation of the downstream pathways.

**Figure 1:**
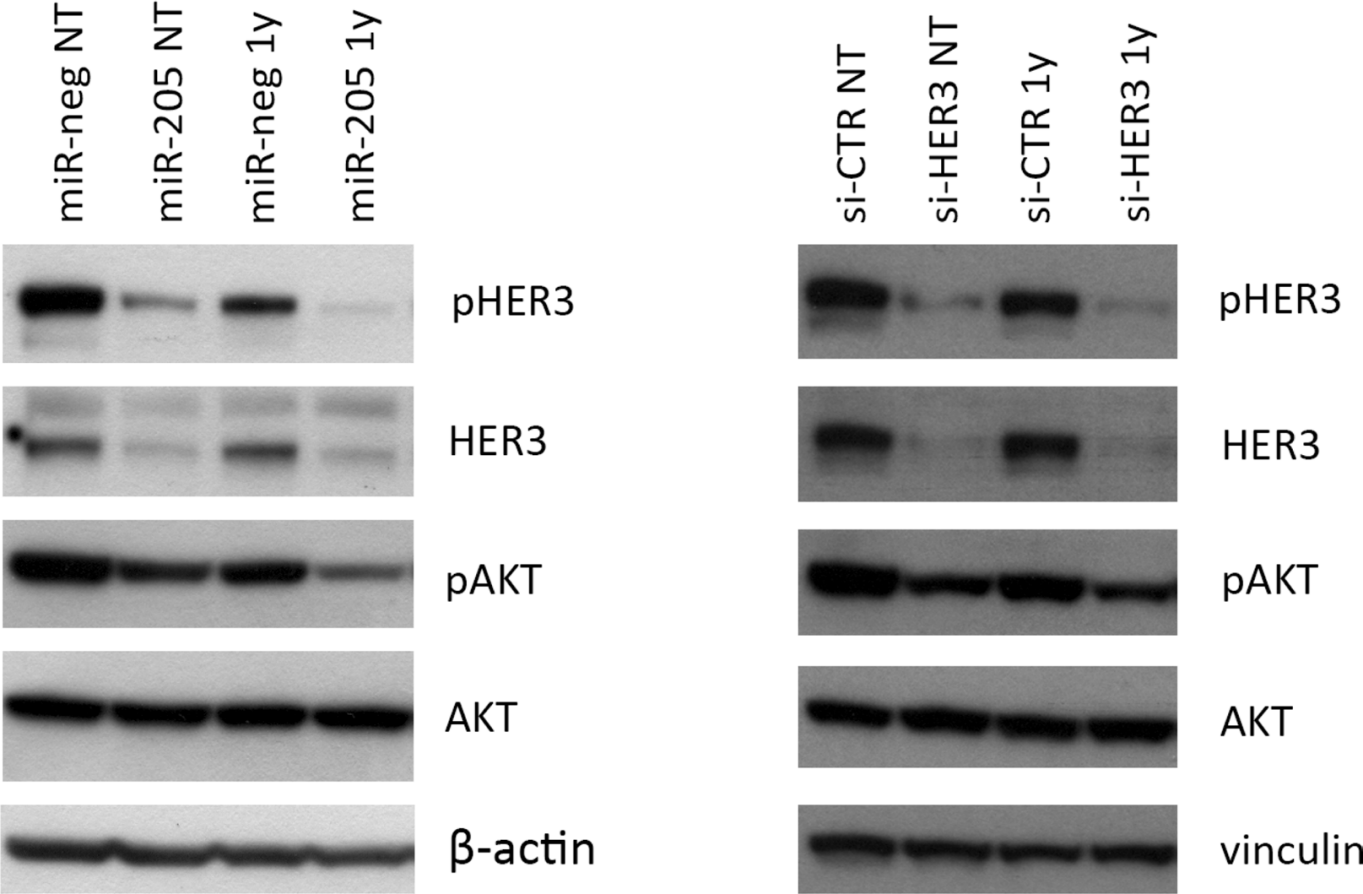
Combined miR-205 and Trastuzumab treatment significantly impairs HER3 activation Combination of Trastuzumab and HER3 silencing, mediated by miR-205 (left panel) or siRNA transfection (right panel), leads to the most significant impairment of HER3 and AKT phosphorylation in HER2+ BC cells. In the left panel, samples have been normalized on β-actin; in right panel samples have been normalized on vinculin. The image is representative of three different experiments.

As shown in Figure 1, the combination of HER3 silencing – either by miR-205 ectopic expression or siRNA-mediated silencing as phenocopy experiment – with trastuzumab treatment leads to the most significant impairment of HER3 activation (measured as p-HER3 levels) and of its downstream mediator AKT.

Similar results were obtained upon ectopic expression of miR-205 in another HER2+ BC cell line (BT474), corroborating the hypothesis that miR-205 can modulate trastuzumab response in the HER2+ subgroup of BC (Supplementary Figure 2).

**Figure 2:**
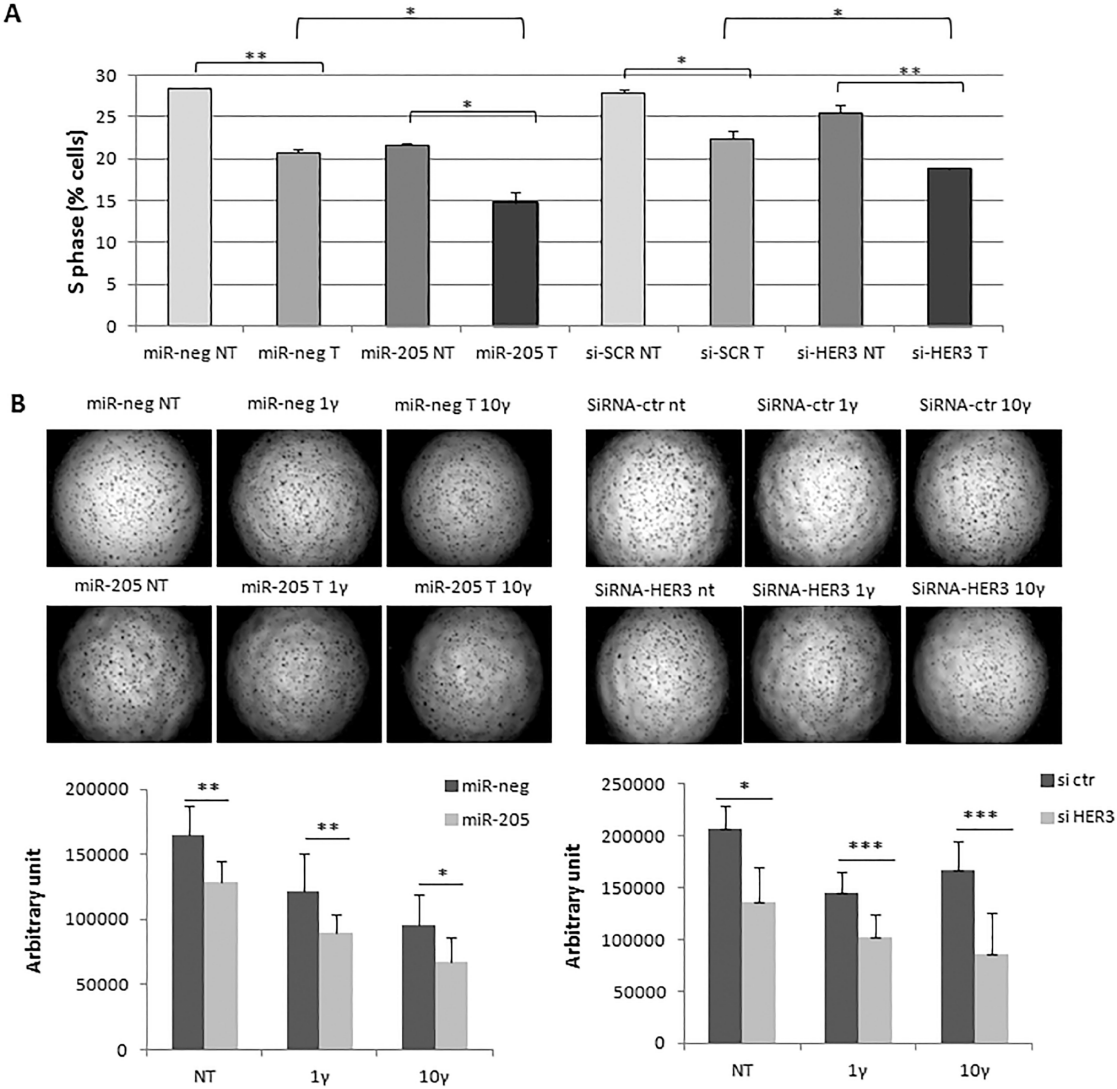
MiR-205 improves responsiveness to Trastuzumab Combination of Trastuzumab and HER3 silencing, mediated by miR-205 or siRNA transfection, leads to the most significant reduction of cell cycle S phase (**A**), and decreased capability to form cell colonies in a 3D matrigel support (**B**). Each image is representative of three different experiments.

To assess the functional effects of miR-205 on Trastuzumab responsiveness, we performed a cell cycle analysis of SKBr3 following 24 h transfection with miR-205 and miR-neg or with si-HER3 and si-CTR, treated for additional 48 h with 2 ug/ml trastuzumab. Results demonstrated a significant reduction in the cell cycle S phase both with miR-205 and with si-HER3 (Figure 2A).

Finally, we performed a 3D colony assay culturing transfected cells with miR-205 and miR-neg or with si-HER3 and si-CTR on matrigel, upon 24 h cells were treated or not with 1 and 10 ug/ml trastuzumab for 72 h. As shown in Figure 2B, the combination of HER3 down-modulation with trastuzumab treatment induces a more significant reduction in the number of colonies.

In summary, miR-205 improves the responsiveness to trastuzumab *in vitro*, and this effect is exerted through impairment of AKT-mediated pathway.

### MiR-205 expression and sensitivity to Trastuzumab in HER2+ BC PDX models

Patient-derived xenografts (PDXs) represent a valid model to study the biology and the heterogeneity of human cancer, and constitute an excellent tool to investigate the responsiveness to specific drugs in a preclinical setting, which strictly recapitulate the features of the human tumors of origin.

**Figure 3:**
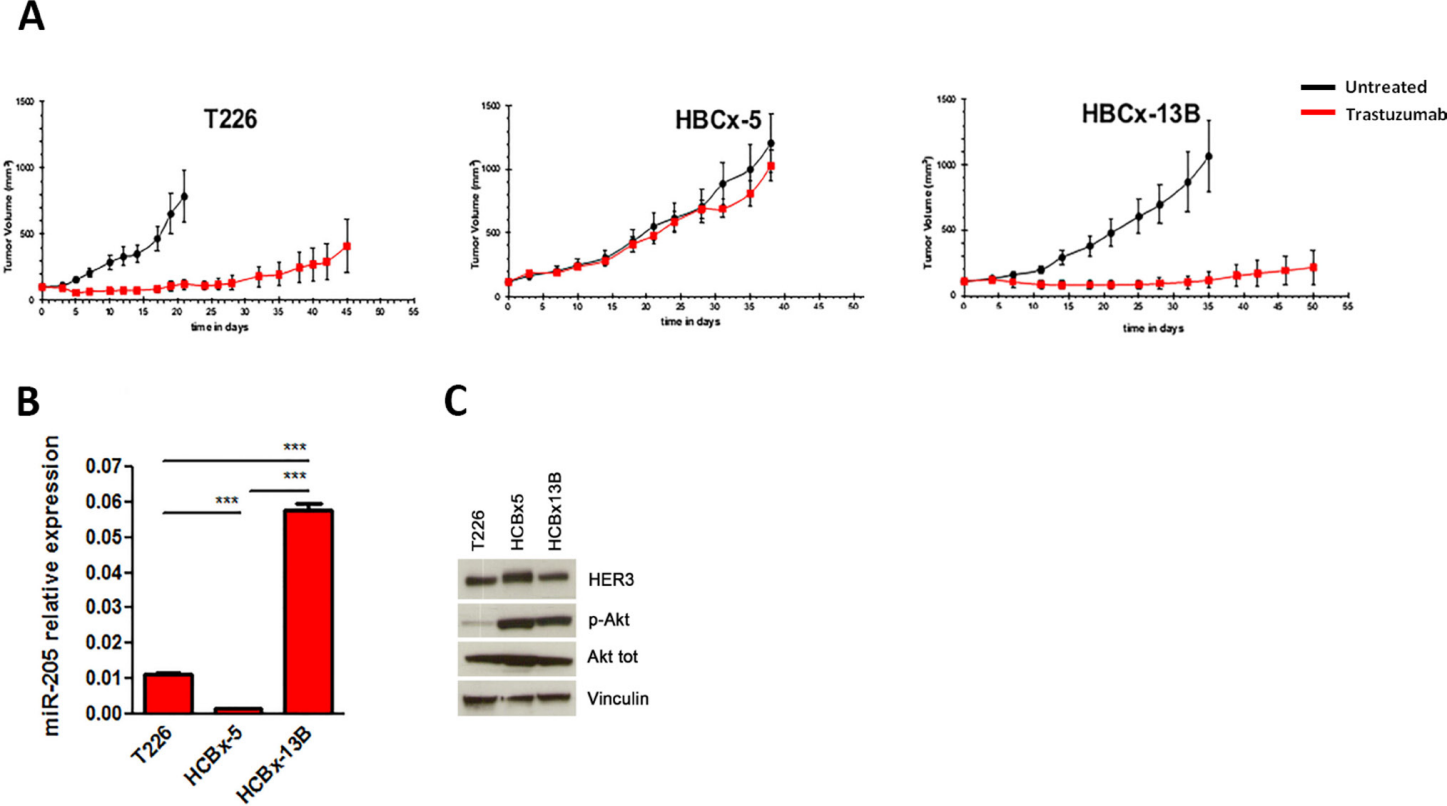
MiR-205 and HER3 expression in PDX models Tumor growth curves of HER2+ BC xenografts implanted on SHO mice as a function of time and treated (red line) or not (dark line) with Trastuzumab (**A**). qRT-PCR showing miR-205 expression in T226, HBCx-5 and HBCx-13B tumors (**B**). Western blot showing HER3, p-AKT and AKT levels (**C**).

Thus, to further confirm the relation between miR-205, HER3 expression and sensitivity to Trastuzumab, we took advantage of HER2+ patient-derived BC xenografts (PDXs), which maintain the characteristics of the primary tumor (Supplementary Figure 3), and show different sensitivity to Trastuzumab (Figure 3A). Interestingly, Trastuzumab-resistant HBCx-5 tumor is characterized by reduced miR-205, higher HER3 and p-AKT levels compared to Trastuzumab-sensitive HBCx-13B and T226 tumors (Figure 3B, 3C), further supporting the idea that loss of miR-205 and the consequent upregulation of its target HER3 are associated to reduced response.

### MiR-205 predicts a favourable outcome in Trastuzumab-treated patients

One of the most crucial and urgent issues in medical oncology is the need of reliable predictive biomarkers, able to efficiently select patients who will most likely benefit from a specific treatment. Resistance to Trastuzumab can be primary or acquired, and defining a tool to monitor the potential tumor responsiveness to the drug and to help treatment decision would be of extreme utility. Since miR-205 directly targets HER3, thus impairing the downstream AKT-mediated pathway and exerting a co-adjuvant effect in combination with Trastuzumab, we also hypothesized that its expression levels could predict the effectiveness of the drug. Thus, to test this hypothesis and investigate the potential role of miR-205 as predictive biomarker of Trastuzumab benefit, we analyzed its expression by qRT-PCR in a series of 52 HER2+ BC patients treated with adjuvant Trastuzumab [18]. MiR-205 expression has been divided in tertiles (Supplementary Figure 4) and, due a similar clinical behaviour, the two curves representing patients expressing low and intermediate levels of the miR-205 have been merged.

**Figure 4:**
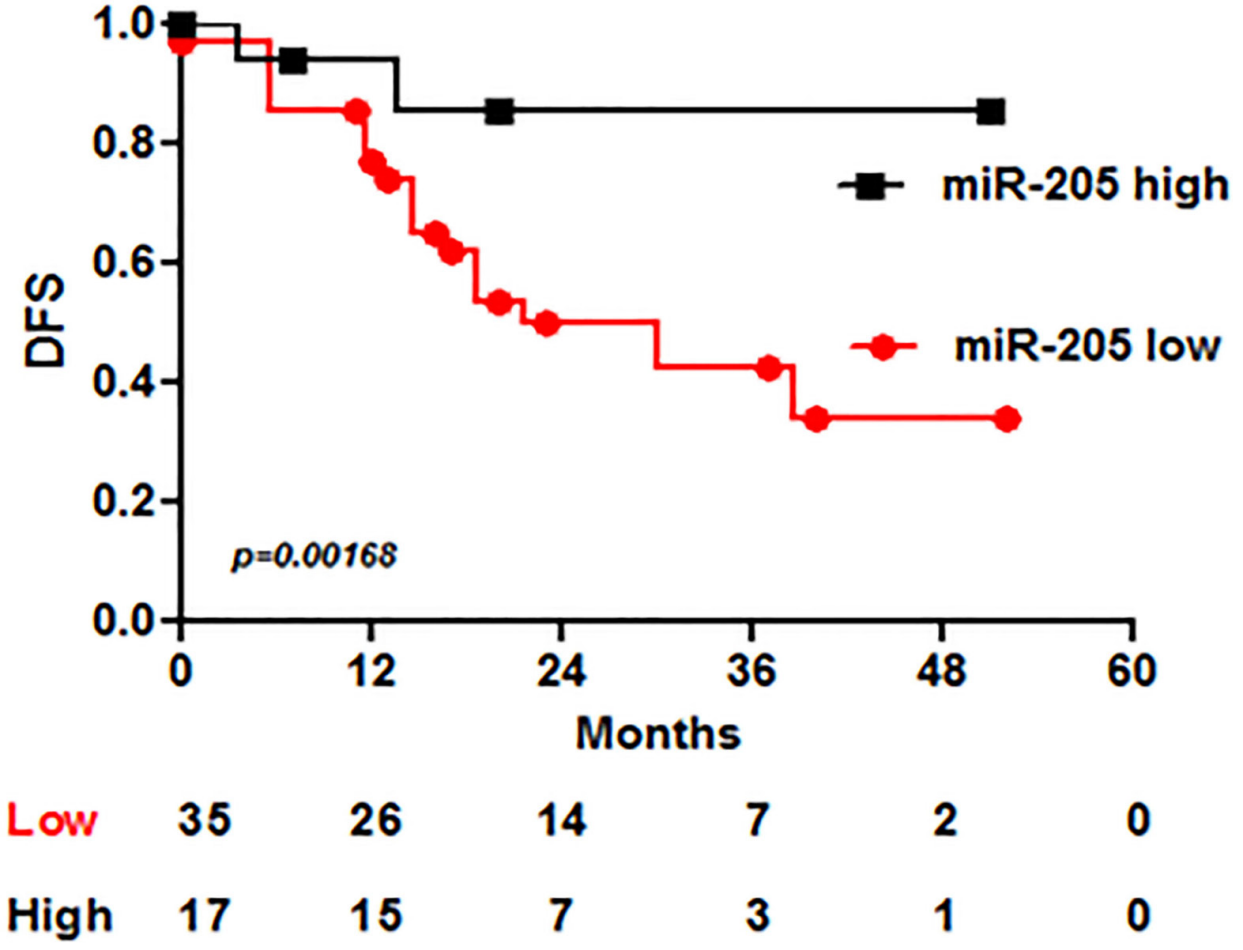
MiR-205 expression associates with DFS in BC patients treated with adjuvant Trastuzumab MiR-205 has been analyzed by qRT-PCR in a set of 52 specimens from HER2+ BC patients treated with Trastuzumab in adjuvant setting. Kaplan-Meyer curve shows the DFS –disease free survival- of patients divided according to miR-205 expression.

We observed that miR-205 expression is significantly associated with outcome: patients with higher miR-205 levels show better DFS (disease-free survival) (*p* = 0.00168) (Figure 4).

## DISCUSSION

Primary or acquired resistance to therapies is a major issue in medical oncology. Several mechanisms have been proposed to justify the resistance to Trastuzumab in eligible patients, however we are still far from having unraveled this matter. It is conceivable that one of the possible escape mechanisms triggered by neoplastic cells to overcome the inhibition of a molecule acting as main driver, as HER2 in BC, is the recruitment and/or hyperactivation of a cognate receptor. Among the HER family members, HER3 is the preferred HER2 partner, indeed the two receptors are frequently co-expressed in HER2+ tumors, and HER3 sustains the tumorigenesis induced by HER2.

Simultaneous inhibition of HER2 and HER3 by combining Trastuzumab with an anti-HER3 antibody significantly improved response to Trastuzumab *in vitro* and *in vivo* [19], and HER3 expression has been correlated with response to the drug [20], even though the data available to date are still controversial.

It is important to underline that the activation of alternative, tumor-intrinsic pathways, are probably not the only escape mechanisms to Trastuzumab treatment: an increasing body of evidence focuses on the role of immune system activation in the responsiveness to the drug, and this is not surprising considering the mechanism of action of the antibody [5]. However, tumor-intrinsic mechanisms and immune infiltration are likely to cooperate in the tentative to dampen the drug efficacy.

On the other side, deregulation of microRNAs is currently a well recognized hallmark of human cancer. Their causal role in the occurrence, progression and resistance to therapies implies the potential use of these small molecules as predictive biomarkers and as therapeutic tools or targets [21], depending on their expression levels and biological function. MiR-205 has generated increasing interest since the first evidence of its involvement in BC biology [22, 23], and our group reported its implication in the direct regulation of HER3 and in the responsiveness to TKI as Gefitinib and Lapatinib [17]. Considering the importance of HER3 in the development of escape mechanisms to anti-HER2 therapies, miR-205 is a good candidate for a potential involvement in the responsiveness to Trastuzumab.

Indeed, here we report that the combination of HER3 silencing, either induced by miR-205 or mediated by a specific siRNA, enhances the response to trastuzumab affecting cell cyle progression and cell growth. *In vitro* data were also confirmed using PDX models, indeed a model of trastuzumab-resistant tumor shows down-regulation of miR-205 and up-regulation of HER3 and p-AKT compared with a trastuzumab-sensitive tumor. Importantly, PDXs generated from tumor patients maintain the same histopathology, tumor behavior, response to therapy and the metastatic properties of the original tumor [24].

Despite the increasing excitement of the scientific community toward a miRNA-based therapy, the potential translation of miRNA delivery into the clinical practice still needs to be proven, and the recent failure of the MIRX34 phase I clinical trial, sponsored by MiRNA Therapeutics and closed in 2016 because of multiple immune-related severe adverse events observed in patients, has raised serious concerns about the safety of these new drugs. However, novel strategies, including different particle formulations and/or engineering with tumor-directed antibody would likely confer higher delivery specificity and reduce side effects. Other trials are indeed on-going with miRNA mimics [25].

Beside the possibility to exploit miRNA modulation with therapeutic purposes, an increasing number of studies have been conducted to define the potential role of microRNAs as prognostic/predictive biomarkers. For HER2+ BC, few microRNAs have been identified associated to trastuzumab response. For example, the overexpression of miR-21 is associated with resistance to neoadjuvant trastuzumab-chemotherapy in HER2-positive BC patients [14], as well as high levels of circulating miR-210 [26].

In our study, miR-205 expression results significantly associated with disease-free-survival in HER2+ BC patients treated in adjuvant with trastuzumab, suggesting that this microRNA could represent a predictive biomarker of anti-HER2 therapy efficacy.

Altogether our findings demonstrate that miR-205, impairing HER3 expression and AKT pathway activation, might represent a new adjuvant therapeutic tool to bypass the resistance to anti-HER2 therapy and a biomarker to predict responsiveness to the treatment.

## MATERIALS AND METHODS

### Patients and samples

The 52 HER2+ tumors analyzed, with a median follow-up of 22 months, were obtained from BC patients treated with adjuvant chemotherapy plus trastuzumab between 2005 and 2009, deriving from our recent multicenter Italian observational study GHEA [18].

Biospecimens used for research consisted of leftover material of samples collected during standard surgical and medical approaches at Fondazione IRCCS Istituto Nazionale dei Tumori of Milan. Samples were donated by patients to the Institutional BioBank for research purposes, and aliquots were allocated to this study after approval by the “Institutional Review Board” and a specific request to the “Independent Ethical Committee” of the Fondazione IRCCS Istituto Nazionale dei Tumori of Milan.

### Cell lines

Human HER2+ BC cell lines SKBr3 and BT474 were purchased from ATCC (Rockville, MD, USA) and cultured in RPMI 1610 medium with 10 % FBS and 1 mM L-glutamine.

Cell lines were obtained between 2000 and 2010, authenticated once a year (last verification on November 2015) using the short tandem repeat profiling method in our Institute facility, and propagated in the suggested media within six months of thawing from stocks.

For transfection experiments, cells were seeded in 6-well plates at 2 × 10^5^/well and transfected with hsa-miR-205 Pre-miR™ miRNA Precursor (Thermo Fisher, Waltham, MA, USA) or Pre-miR™ Negative Control #1 (Thermo Fisher, Waltham, MA, USA). For gene knock down, specific siRNAs (Thermo Fisher, Waltham, MA, USA) were used. Cells were incubated with 100 nM miRNA precursors or 50 nM siRNAs complexed with Lipofectamine 2000 transfection reagent (Thermo Fisher, Waltham, MA, USA) according to manufacturer's instructions.

### RNA extraction and quantitative RT-PCR

Total RNA was extracted from cell lines with Qiazol reagent (Qiagen, Valencia, CA, USA). For miRNA quantification, cDNA was synthesized from 100 ng of RNA with TaqMan MiRNA Reverse Trascription Kit (Thermo Fisher, Waltham, MA, USA). QRT-PCR was performed using TaqMan assays for human miR-205*, RNU44* and *RNU48* (Thermo Fisher, Waltham, MA, USA).

MiRNA levels were normalized to the endogenous control *RNU44* or *RNU48* and the relative expression was calculated using the comparative 2^-ΔCt method.^

### Western blot

Total protein lysates were extracted with lysis buffer (1% Triton, 50 nM Tris, 15 mM NaCl) supplemented with protease inhibitors (Sigma-Aldrich, St. Louise, MO, USA). The following primary antibodies were used: rabbit anti-human IgG antibodies against pHER3 (21D3, #4791), pAKT (D9E, #4060), AKT (#9272) (Cell Signaling, Danvers, MA, USA), HER3 (C-17, sc-285) (Santa Cruz, Dallas, Texas, USA); Vinculin (V9131); mouse horseradish peroxidase-conjugated anti-human IgG anti-β actin (A3854) (Sigma-Aldrich, St. Louise, MO, USA). Proteins were visualized by enhanced chemiluminescence detection system (Sigma-Aldrich, St. Louise, MO, USA). Quantification was performed by Quantity One 4.6.6 software (Bio-Rad, Hercules, CA, USA).

### Cell cycle analysis

SKBr3 were transfected with 100 nM hsa-miR-205 Pre-miR™ miRNA Precursor (Thermo Fisher, Waltham, MA, USA) or Pre-miR™ Negative Control #1 (Thermo Fisher, Waltham, MA, USA) for 24 h and treated with 2 μg Trastuzumab for additional 48 h. Cells were then trypsinized, washed in PBS and fixed in 70% ethanol for 2 h at −20° C. After fixation, cells were washed in PBS and centrifuged for 5 min at 1200 rpm. Cells were resuspended in PBS containing 10 mg/ml propidium iodide (Roche, Basel, Switzerland) and incubated for 20 min at 37° C. Cells were analyzed using FACS-Calibur flow cytometer and the results were further analyzed with the ModFit software, v3.2 (Verity Software House).

### 3D colony assay

SKBr3 were transfected with 100 nM hsa-miR-205 Pre-miR™ miRNA Precursor (Thermo Fisher, Waltham, MA, USA) or Pre-miR™ Negative Control #1 (Thermo Fisher, Waltham, MA, USA) and 50 nM siRNA HER3 or siRNA negative control. Following 24 h of transfection, cells were seeded in 96 wells coated with matrigel (Corning, New York, NY, USA) at a density of 5 × 10^3^ cells/well and treated or not with 1 μg and 10 μg trastuzumab for 5 days. Images of colony formation were acquired using a EVOS inverted light microscope, and quantified by a macro made with the Image-Pro Plus 7.0 software. The mean ± S.D. of three replicates is reported.

### Patient-derived BC xenografts (PDXs)

PDXs from HER2 positive patients (T226, HBCx-5 and HBCx-13B) were obtained in collaboration with Xentech (Evry, Paris). Efficacy studies to assess the sensitivity to Trastuzumab were performed in compliance with the required ethical standards. Briefly, BC fragments were obtained from patients at the time of surgery, with informed written patient consent, and solid tumor xenografts engrafted in SCID Hairless Outbred (SHO) mice (Charles River Laboratories, L’Arbresle, France).

Tumor xenografts were allowed to reach a volume of ~75–150 mm^3^ before randomizing the mice into groups of 8 to 10 based on tumor size. Tumors were measured two or three times a week after initiation of treatment, and volumes were determined using the formula: volume = (width^2^ × length)/2. Animals were treated with trastuzumab (10 mg/kg) by intra-venous or i.p. injection biweekly, for 6 weeks.

Every procedure has been previously approved by the Ethical Committee CE51 at CERFE animal facility at GENOPOLE, Evry, France.

### Statistics

Data are expressed as mean ± s.d. Statistical comparisons were tested by Student's *t*-test using Graphpad Prism software. *P* < 0.05 was considered statistically significant.

To evaluate the association of miR-205 expression with outcome in BC patients, human samples were divided in tertiles according to the miRNA expression, as assessed by qRT-PCR.

## SUPPLEMENTARY MATERIALS AND FIGURES



## Abbreviations

BC: breast cancer
TKI: tyrosin kinase inhibitor
PR: pathological response
PDX: patient-derived xenograft
