# Supplementary material for: MiR-205 as predictive biomarker and adjuvant therapeutic tool in combination with trastuzumab
Source: Oncotarget. 2018 Jun 15;9(46):27920–8. doi: 10.18632/oncotarget.24723 (PMC6021348; doi:10.18632/oncotarget.24723)
Supplement: Supplementary file 1 [file oncotarget-09-27920-s001.pdf]

MiR-205 as predictive biomarker and adjuvant therapeutic tool in combination with trastuzumab

SUPPLEMENTARY MATERIALS

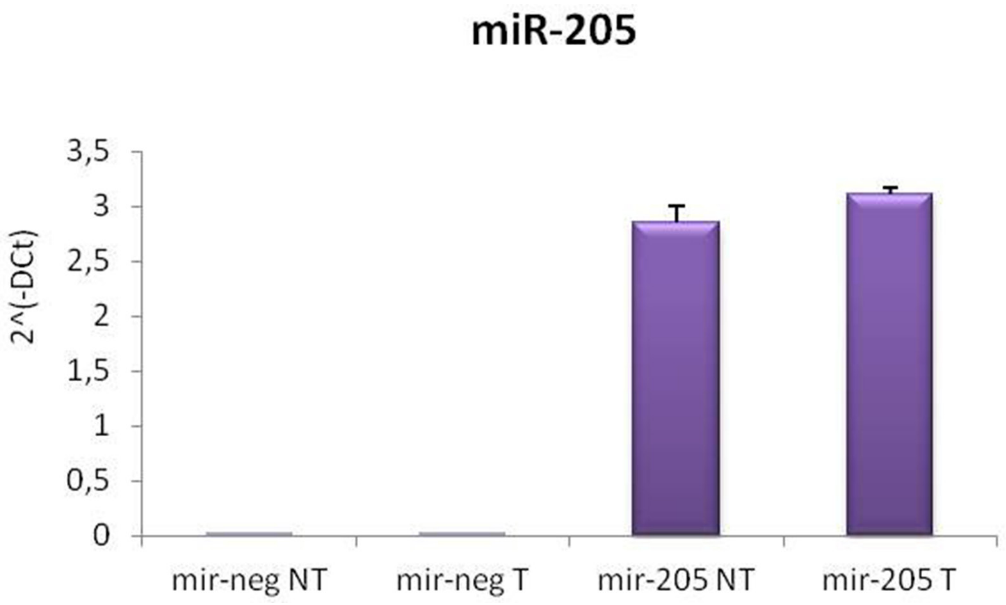

Supplementary Figure 1: Evaluation of the transfection efficiency by qRT-PCR analysis of miR-205 levels in transfected versus control cells.

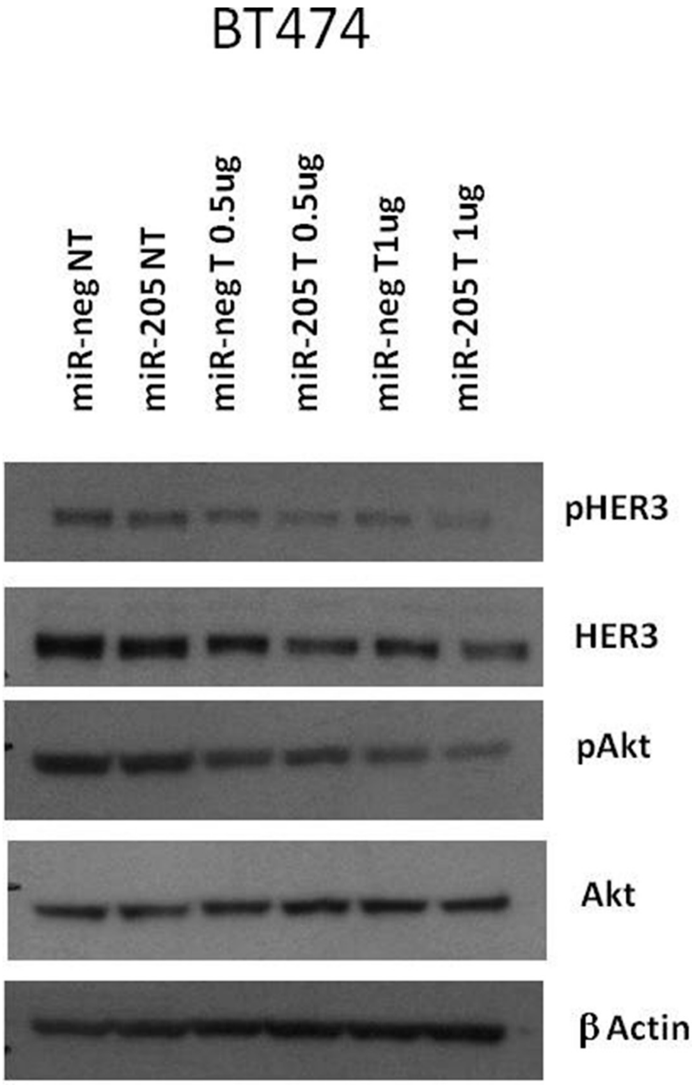

Supplementary Figure 2: Evaluation of p-HER3 and p-AKT reduction following combination of miR-205 transfection and trastuzumab treatment in HER2+ BC cell line BT474.

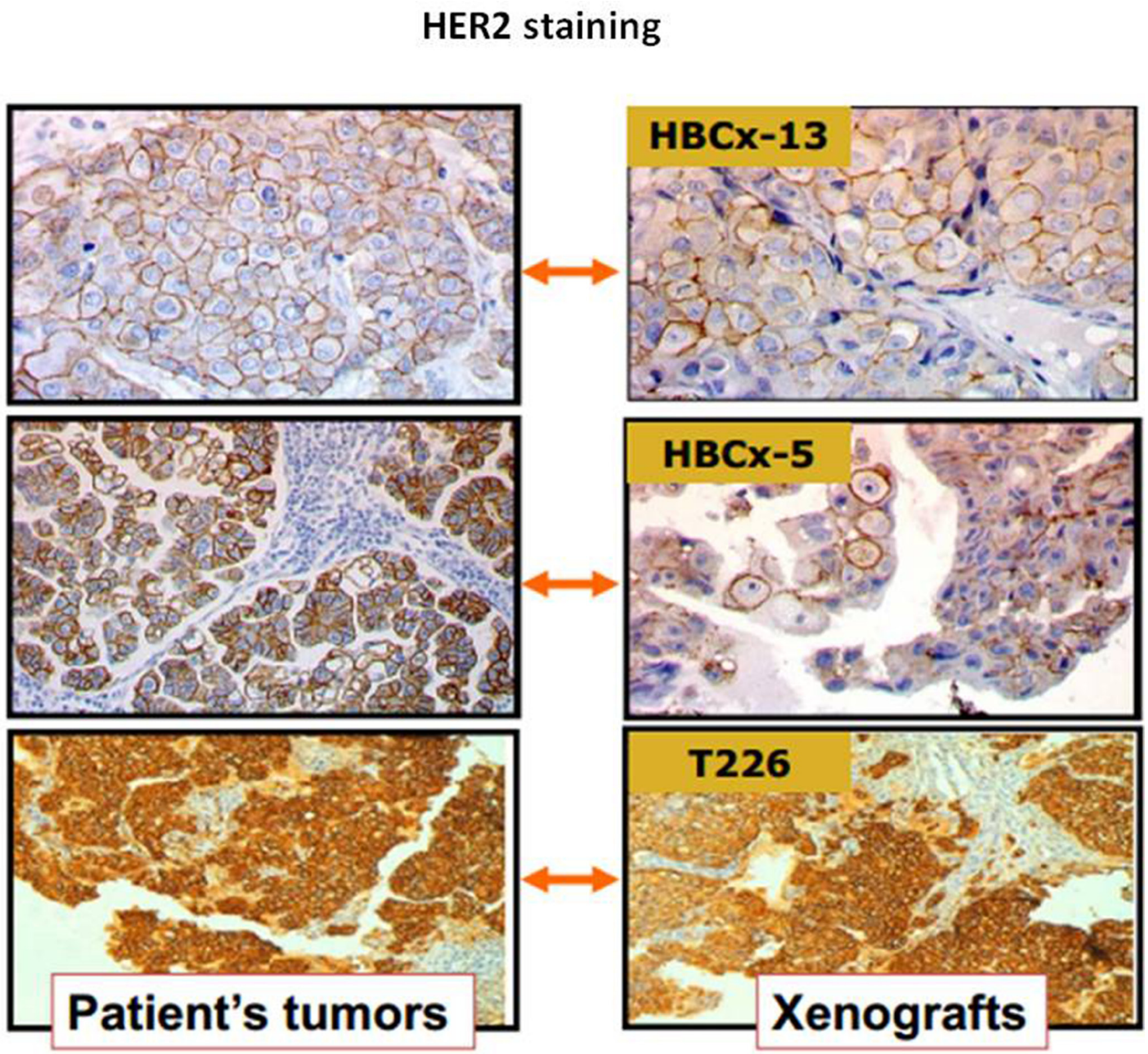

Supplementary Figure 3: Histology and immunostaining of BC patient-derived xenografts with antibodies directed against HER2, showing conservation of histological features and HER2 expression in comparison with the primary tumor.

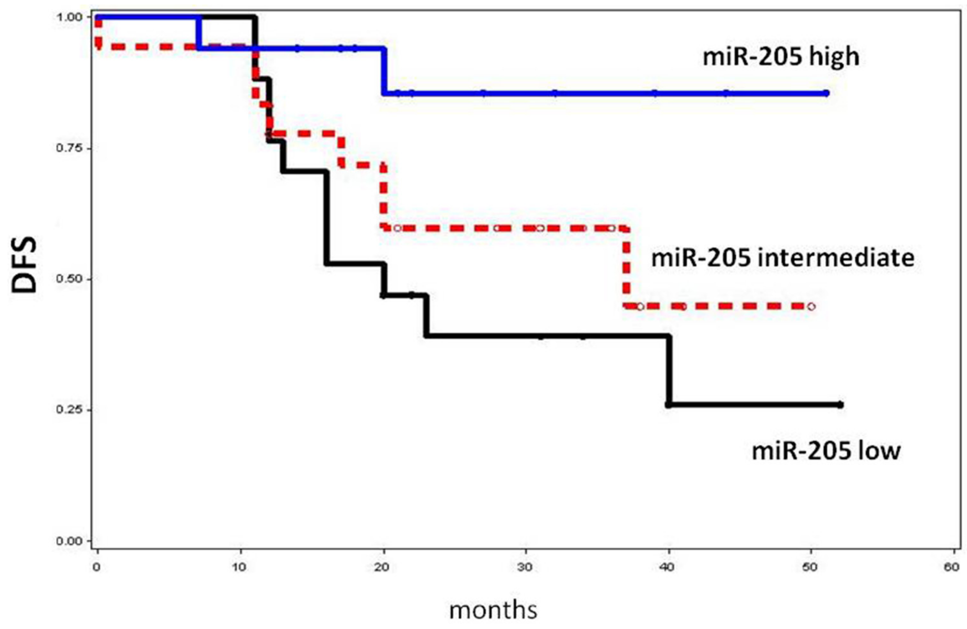

Supplementary Figure 4: Kaplan–Meyer curves showing the different DFS (disease-free survival) of patients affected with HER2 positive BC and treated with adjuvant Trastuzumab, stratified in tertiles according to miR-205 expression.
